# Possible role of zinc (Zn) as an adjunctive therapy in gastrointestinal symptoms of COVID-19 infectious disease 

**Published:** 2020

**Authors:** Mohammad Abbasinazari

**Affiliations:** *Department of Clinical Pharmacy, School of Pharmacy, Shahid Beheshti University of Medical Sciences, Tehran, Iran *

**To The Editor**

 The novel coronavirus (COVID-19) disease is caused by SARS-CoV-2, which is the causative agent of a high mortality disease that is of great concern to world public health. The coronavirus belongs to the largest family of RNA viruses, meaning it is a single-stranded enveloped RNA virus possessing a positive-sense RNA genome with a 5′-capstructure and 3′-poly-A tail (1). RNA viruses have evolved into a variety of replication strategies, but they are unified in the fact that an RNA-dependent RNA polymerase (RdRp) functions as the main enzyme of their RNA synthesizing machinery. The RdRp is commonly embedded in a membrane-associated replication complex that is assembled from viral RNA and viral and host proteins. At present, RdRps are one of the key targets for the development of antiviral medications. An increased level of intracellular Zinc (Zn) causes the efficiently impaired replication of a number of RNA viruses by interfering with the correct proteolytic processing of viral polyproteins (2). In vitro studies have reported that coronavirus replication can be inhibited by increased Zn concentration (2).

A significant proportion of COVID-19 patients can present initially with only gastrointestinal (GI) symptoms. The most common GI symptoms are anorexia, nausea, vomiting, and diarrhea. Currently there is no definite evidence to suggest that the severity of GI symptoms corresponds to the severity of the COVID-19 clinical course. There is no specific treatment for GI symptoms in COVID-19 infected patients, but supportive measures are recommended (3). The single layer of epithelial cells in the mucosa of the GI tract, held together by tight junctions, provides a barrier between the external environment and the body. Zn is considered a key factor in the preservation of the structural integrity of the intestinal barrier. Every mechanism, every stress that breaches the integrity of the GI barrier, may modify the state of health of the GI mucosa and have biological and clinical consequences. In addition to inhibiting viral replication, Zn may halt the progression of the GI disease by its participation in free radical scavenging, halting the inflammatory process (4). 

Although Zn is the fourth prevalent metal in the world, Zn deficiency is a subject of health concern in both developing and developed countries, particularly among infants and the elderly (5). Zn homeostasis, both extra- and intracellular, indicates that it plays an important role in human immunity. Although Zn is a component of about 10% of the human proteome, Zn in different forms (free and protein bound) can stimulate a variety of signaling events, including the antiviral response (6). Despite the important role of Zn in immune function, Zn deficiency has been reported in different viral diseases. In a cross-sectional study, Zn levels in patients with cirrhosis induced by either hepatitis B or C were investigated. This study concluded that the Zn levels of evaluated patients were lower than half the normal range (7).

With attention to both antiviral and immune enhancement, several clinical studies have shown the efficacy and safety of Zn supplementation in the treatment of infectious diseases induced by RNA viruses. A meta-analysis of randomized controlled trials reported the positive role of oral Zn (range from 80-92 mg/day of elemental Zn) in the alleviation of common cold symptoms. Among the patients studied, 70% of those who received Zn had recovered by the fifth day compared with 27% of the placebo patients. In addition, none of the studies observed serious adverse effects with Zn (8). In another meta-analysis, Zn supplementation had a significant effect on the sustained viral response (SVR) during combination therapy with interferon and ribavirin of patients with chronic hepatitis C infection (9).

There is no published randomized clinical trial investigating the effects of Zn in alleviating or managing the GI symptoms of patients infected with COVID-19, but on the basis of in vitro studies, Zn supplementation may affect not only COVID‐19‐related symptoms in respiratory tract infections, but also COVID‐19 itself. 
